# Postmortem Human Dura Mater Cells Exhibit Phenotypic, Transcriptomic and Genetic Abnormalities that Impact their Use for Disease Modeling

**DOI:** 10.1007/s12015-022-10416-x

**Published:** 2022-07-09

**Authors:** Andrea R. Argouarch, Nina Schultz, Andrew C. Yang, Yeongjun Jang, Kristle Garcia, Celica G. Cosme, Christian I. Corrales, Alissa L. Nana, Anna M. Karydas, Salvatore Spina, Lea T. Grinberg, Bruce Miller, Tony Wyss-Coray, Alexej Abyzov, Hani Goodarzi, William W. Seeley, Aimee W. Kao

**Affiliations:** 1 Memory and Aging Center, Weill Institute for Neurosciences, Department of Neurology, University of California San Francisco, San Francisco, CA 94158, USA; 2 Department of Neurology and Neurological Sciences, School of Medicine, Stanford University, Stanford, CA 94304, USA; 3 Department of Quantitative Health Sciences, Center for Individualized Medicine, Mayo Clinic, Rochester, MN 55905, USA; 4 Department of Biochemistry and Biophysics, University of California San Francisco, San Francisco, CA 94158, USA; 5 Department of Urology, University of California San Francisco, San Francisco, CA 94158, USA; 6 Department of Pathology, University of California San Francisco, San Francisco, CA 94158, USA

**Keywords:** Human dura mater, Dermal epithelium, Dural cells, Dermal fibroblasts, Mural cells, Postmortem tissue, Chromosomal karyotype, Loss of Y chromosome, Biobanking, Neurodegenerative disease

## Abstract

Patient-derived cells hold great promise for precision medicine approaches in human health. Human dermal fibroblasts have been a major source of cells for reprogramming and differentiating into specific cell types for disease modeling. Postmortem human dura mater has been suggested as a primary source of fibroblasts for in vitro modeling of neurodegenerative diseases. Although fibroblast-like cells from human and mouse dura mater have been previously described, their utility for reprogramming and direct differentiation protocols has not been fully established. In this study, cells derived from postmortem dura mater are directly compared to those from dermal biopsies of living subjects. In two instances, we have isolated and compared dermal and dural cell lines from the same subject. Notably, striking differences were observed between cells of dermal and dural origin. Compared to dermal fibroblasts, postmortem dura mater-derived cells demonstrated different morphology, slower growth rates, and a higher rate of karyotype abnormality. Dura mater-derived cells also failed to express fibroblast protein markers. When dermal fibroblasts and dura mater-derived cells from the same subject were compared, they exhibited highly divergent gene expression profiles that suggest dura mater cells originated from a mixed mural lineage. Given their postmortem origin, somatic mutation signatures of dura mater-derived cells were assessed and suggest defective DNA damage repair. This study argues for rigorous karyotyping of postmortem derived cell lines and highlights limitations of postmortem human dura mater-derived cells for modeling normal biology or disease-associated pathobiology.

## Introduction

The appeal of human in vitro cell models stems from the potential to recapitulate and elucidate the pathophysiology of numerous diseases, including neurodegenerative disorders. Patient-derived cell lines can address cellular and molecular pathways that complement other experimental model systems, such as stress, autophagy, and trafficking [1-3]. The utility of patient-derived cell models depends upon their ability to reliably reflect normal or pathophysiological processes. For example, a fibroblast patient-derived cell model of Huntington’s disease has been reported to clinically recapitulate the disease state better than transformed cellular models [4]. In this regard, fibroblasts isolated from dermal biopsies are now routinely collected in order to be directly differentiated into induced neurons (iNeurons) or reprogrammed into induced pluripotent stem cells (iPSCs) [5, 6] that can subsequently be differentiated into many different cell types. Further incorporation of induced neurons into more complex cellular models, such as three-dimensional brain organoids, represents additional avenues to replicate morphological and functional cell interactions of the human brain [7].

Fibroblasts from dermal biopsies performed on living research subjects have been a common cell source for patient-derived disease models. Fibroblasts can be isolated at various stages during life (presymptomatic, symptomatic, and advanced disease) and thus can potentially be used to model different phases of disease progression. In certain circumstances, however, tissues are not collected during life. In these instances, it would be advantageous to access fibroblasts from postmortem tissue, such as when family members who carry relevant gene mutations are identified or when no other sources are available. For these reasons, an accessible source of postmortem fibroblasts from patients could provide significant value to the field.

Human dura mater can provide an alternative source of primary cells that can be directly obtained from postmortem tissue. Dura mater is the outer most layer of the meninges, located between the skull and the brain, and is primarily composed of fibroblasts and collagen [8]. Cells from dura mater can be isolated, cultured, and utilized for several applications. For example, previous studies have investigated the use of dura mater-derived cells in grafts [9], drug response [10, 11], particle engulfment [12, 13], single nucleotide variant identification [14], inflammation and healing [15, 16], and reprogramming into iPSCs [17-19]. Brain biobanks oftentimes collect and store dura mater at the time of autopsy. The use of these dura mater-derived cells can be advantageous because they can be studied when other cell sources, such as dermal fibroblasts or peripheral blood mononuclear cells, are not otherwise available. Although dura mater-derived cells have been used in multiple studies, a direct comparison between dermis and dura mater-derived cells has not been reported to our knowledge.

In this study, we endeavored to characterize cells isolated from postmortem human dura mater to assess their utility as a source of cells for in vitro disease modeling. Others have previously isolated dura mater-derived cells from non-cryoprotected frozen dura mater [19]. Similarly, we isolated cells from postmortem frozen and fresh dura mater from subjects with neurodegenerative diseases and compared them to traditionally isolated dermal fibroblasts. Features compared included cell growth and morphology, chromosomal karyotype, expression of fibroblast and mural markers, RNA expression profiles, and mutation signatures from whole genome sequencing (WGS). In two cases, we were able to compare dermal fibroblasts and postmortem dura mater-derived cells from the same subject. This direct, comparative analysis between cells isolated from the dermis and dura mater highlights the potential limitations of postmortem dura mater-derived cells for in vitro personalized medicine applications and disease modeling.

## Materials and Methods

### Tissue Collection

Dermal tissue was provided by the University of California, San Francisco, Memory and Aging Center, where participants enrolled in observational research studies underwent skin biopsy after providing written informed consent. Approximately two-millimeter dermal punches were performed at the inner thigh. The tissue was immersed in complete media composed of Dulbecco’s Modified Eagle Medium (DMEM) high glucose with sodium pyruvate (Thermo Fisher Scientific, Waltham, MA, USA, #11995073), 10% heat inactivated fetal bovine serum (FBS) (VWR, Radnor, PA, USA, #97068-091), and 1% Penicillin-Streptomycin (Thermo Fisher Scientific, #15140122). Cells were isolated within eight hours after procurement.

Postmortem human dura mater was provided by the University of California, San Francisco, Neurodegenerative Disease Brain Bank. Consent for brain donation was obtained from all subjects or their surrogates, following the principles outlined in the Declaration of Helsinki. Prior to brain extraction, the overlying dura mater was removed and stored surrounded by wet ice during transport. Upon arrival to the laboratory, a small patch of dura mater ranging in maximum dimension from two to five centimeters, was dissected and either (1) immersed in complete DMEM media, described above, in a centrifuge tube or (2) rapidly frozen and banked. The freshly captured dural tissue was stored at 4 °C, and cells were isolated within 24 hours after procurement. For frozen dural tissues, samples were placed in a freezer bag and surrounded by dry ice, then stored in a − 80 °C freezer for up to 4 years of long-term storage without the use of a cryoprotectant reagent. The dural tissue was briefly thawed and cells were isolated [19].

### Cell Culture

Following tissue collection, both dermal and dural tissues were washed in sterile Dulbecco’s phosphate buffered saline (DPBS) without calcium and magnesium (Thermo Fisher Scientific, #14190250) four times, one minute per wash, in a cell culture dish. The tissue was then transferred into a new dish with complete DMEM media. The tissue was divided into smaller sections, approximately one to two millimeters, with surgical tools and transferred into a six-well cell culture plate. A glass coverslip was gently placed over the tissue and complete DMEM media was added into the wells. For the remaining fresh dural tissue, it was cryopreserved in complete DMEM media and 10% dimethyl sulfoxide (DMSO) for long-term tissue storage in liquid nitrogen. Cultures were incubated in a humidified chamber at 37 °C and 5% CO_2_, left undisturbed for the first week, then fed every two to three days with complete DMEM media. After approximately three weeks in culture, the cells were collected, expanded, and banked in liquid nitrogen with complete DMEM media and 10% DMSO [20].

To optimize the recovery of cells from frozen dural tissue, an alternative cell culture media was used, composed of DMEM high glucose with sodium pyruvate, 10% heat inactivated FBS, 1X Non-Essential Amino Acid (NEAA) (Thermo Fisher Scientific, #11140050), 1X Glutamax (Thermo Fisher Scientific, #35050061), 0.1 mM 2-Mercaptoethanol (Thermo Fisher Scientific, #21985023), 1X Nucleosides (MilliporeSigma, Burlington, MA, USA, #ES-008-D), and 1% Penicillin-Streptomycin [18, 19]. This DMEM media with nucleosides was used for the first week in culture and then switched to complete DMEM media with the addition of Glutamax and 2-Mercaptoethanol. Alternatively, DMEM media with nucleosides was used throughout the entire time in culture.

### Immunofluorescence

Cell lines were cultured and plated in either a four-well (Thermo Fisher Scientific, #177437) or eight-well (Ibidi GmbH, Munich, Germany, #80826) cell culture chamber slide. Cell lines included either dermis and frozen dura mater-derived cell lines from the same subject and/or a fresh dural cell line. Upon confluency, cells were washed twice with DPBS with calcium and magnesium (MilliporeSigma, #D8662), fixed with 4% paraformaldehyde (Electron Microscopy Sciences, Hatfield, PA, USA, #15714) for 20 minutes, washed with DPBS^+/+^ three times, and then permeabilized with 0.1-0.4% Triton X-100 (MilliporeSigma, #T8787) for 10 or 15 minutes. Cells were washed with DPBS^+/+^ and blocked in 1% bovine serum albumin (BSA, Thermo Fisher Scientific, #BP1605-100) with 0.25% Triton X-100 for 30 minutes or 10% normal goat serum (Abcam, Cambridge, United Kingdom, #ab7481) with 0.1% Triton X-100 for one hour. The following primary antibodies were used: Rabbit anti PDE3A (Phosphodiesterase 3A, 2 μg/ml, Thermo Fisher Scientific, #PA5-82503), Rabbit anti CD146 (1:50, Thermo Fisher Scientific, #17564-1-AP) in block solution overnight at 4 °C, and rhodamine phalloidin conjugated to tetrameth-ylrhodamine (TRITC, Thermo Fisher Scientific, #R415) in block solution for one hour. The cells were washed with DPBS^+/+^ three times and then incubated with Alexa Fluor 488 Goat anti Rabbit (1:250, Thermo Fisher Scientific, #A-11008) in block solution for one hour. Cells were washed with DPBS^+/+^ three times, stained with 4′,6-diamidino-2-phenylindole (DAPI, 300 nM, Thermo Fisher Scientific, #D1306) for five minutes and washed for a final time. All solutions were made in DPBS^+/+^ and performed at room temperature, unless stated otherwise. Alternatively, slides were mounted with Prolong Gold Antifade Reagent with DAPI (Cell Signaling Technologies, Danvers, MA, USA, #8961). Cells were imaged on the DMi8 confocal platform (Leica Microsystems, Wetzlar, Germany) with a 10x objective.

### Cell Proliferation

Dermis and dura mater-derived cell lines were plated at the same seeding density in multiple 24-well cell culture plates (5000 cells per well) at day 0 [21]. Over the span of six days, cells were counted every day to assess proliferation. Cells were washed with DPBS^−/−^, trypsinized with 0.05% Trypsin-EDTA (Thermo Fisher Scientific, #25300062), and collected for cell counting. Cell counts were determined utilizing the Bio-Rad TC20 automated cell counter (Bio-Rad Laboratories, Hercules, CA, USA, #1450102) with a 1:1 dilution of trypan blue solution (Thermo Fisher Scientific, #15250061) to cell suspension. Cell counts were performed in triplicates.

For the Click-it EdU (5-ethynyl-2′ -deoxyuridine) imaging assay, paired dermis and dura mater-derived cell lines from the same subject were plated and stained according to the manufacturer’s instructions. Briefly, a final concentration of 10 μM EdU was added to complete DMEM media and the cells were incubated for one hour in normal cell culture conditions. Cells were fixed with 3.7% paraformaldehyde for 15 minutes, washed with DPBS^+/+^ with 3% BSA twice, and then permeabilized with 0.5% Triton X-100 for 20 minutes. The cells were washed with DPBS^+/+^ with 3% BSA twice, incubated with the Click-iT Plus reaction cocktail for 30 minutes at room temperature, washed twice, counterstained with DAPI (300 nM) for five minutes, and then washed for a final time. Cells were imaged on the DMi8 confocal platform (Leica Microsystems, Wetzlar, Germany) with a 10x objective.

### Chromosomal Karyotyping and Mycoplasma Analysis

For karyotype analysis, dermis and dura mater-derived cell lines were cultured in a T-25 cell culture flask and shipped live to Cell Line Genetics (Madison, WI, USA) for karyotype analysis using G-banding [22]. For mycoplasma detection, cells were collected, washed with DPBS^−/−^, and DNA was extracted using the QuickExtract DNA Extraction Solution (Epicentre, Madison, WI, USA, #QE09050) according to the manufacturer’s instructions. Mycoplasma PCR detection was performed according to the manufacturer’s instructions (Bulldog Bio, Portsmouth, NH, USA, #25234).

### Western Blot

Dermis and dura mater-derived cell lines were collected, washed with DPBS^−/−^, and lysed in cold RIPA buffer (Thermo Fisher Scientific, #89900) with protease (MilliporeSigma, #4693124001) and phosphatase (MilliporeSigma, #4906837001) inhibitors. Lysates were spun and the supernatants were collected for western blot analysis. Protein concentration was determined by bicinchoninic acid assay according to the manufacturer’s instructions (Thermo Fisher Scientific, #23225). Samples were denatured (20 μg each), separated on a NuPAGE 4-12% Bis-Tris protein gel (Thermo Fisher Scientific, #NP0336BOX), and transferred onto a nitrocellulose membrane. Membranes were blocked in Odyssey blocking buffer (LI-COR, Lincoln, NE, USA, #927-50000) for one hour at room temperature, incubated with primary antibodies overnight at 4 °C, and incubated with the appropriate LI-COR secondary antibodies for one hour at room temperature. Membranes were imaged on the LI-COR Odyssey CLx imaging system. The following primary antibodies were used: Rabbit anti-Vimentin (1:1000, Cell Signaling Technologies, #5741S), Rabbit anti-S100A4 (1:1000, Cell Signaling Technologies, #13018S), and Mouse anti-Actin (1:5000, MilliporeSigma, #MAB1501). The following secondary antibodies were used: 680RD Donkey anti-Rabbit (1:10,000, LI-COR, #926-68073), 800CW Donkey anti-Rabbit (1:10,000, LI-COR, #926-32213), and 800CW Donkey anti-Mouse (1:10,000, LI-COR, #926-32212).

### RNA-Sequencing

From the same subject, fresh dermal and frozen dural cell lines were plated into a six-well cell culture plate and isolated for RNA-sequencing. Cells were trypsinized, washed with PBS^−/−^, and total RNA was extracted and purified using the Quick-RNA Microprep Kit (Zymo Research, Irvine, CA, USA, #R1051). Samples were extracted in quadruplicates and RNA was quantified using a NanoDrop. Libraries for RNA-sequencing were prepared using the SMARTer Stranded Total RNA-Seq Kit v2 Pico Input Mammalian library preparation protocol (Takara Bio USA, Inc., Mountain View, CA, USA, #634413), analyzed on Agilent TapeStation 4200 (Agilent Technologies, Inc., Santa Clara, CA, USA, #G2991AA), and pooled before paired-end 150-base-pair sequencing on one lane of an Illumina NovaSeq 6000 sequencer (Illumina, Inc., San Diego, CA, USA), which generated 3 million reads per sample. Salmon (v0.13.1) was used to map reads to the human transcriptome (Gencode v28). Tximport (v1.14.0) was used to import the data into R (v3.6.1) and DESeq2 (v1.26.0) was then used to perform differential gene expression analysis. For predicting enriched pathways, Enrichr [23] or Metascape [24] was used with *Homo sapiens* set as input species. The human vascular single-nuclei RNA-sequencing data object was downloaded [25] with cell type annotations, and analyzed/visualized using Seurat’s [26] FeaturePlot and DotPlot functions to map the cell type-specific expression of top-upregulated frozen dural cell line genes.

### Whole Genome Sequencing

From the same subject, fresh dermal and frozen dural cell lines were cultured into a cell culture flask, trypsinized, washed with DPBS^−/−^, and shipped to GENEWIZ, Inc. (South Plainfield, NJ, USA) for WGS. Bulk DNA was isolated by Qiagen QIAmp DNA Kit and HT DNA Kit. DNA was quantified using the Qubit 2.0 Fluorometer (Thermo Fisher Scientific) and the DNA integrity was verified with ~1% agarose gel. Libraries were prepped with NEBNext Ultra DNA Library Prep Kit and paired-end 2x150-base-pair sequencing was performed with the Illumina HiSeq (Illumina, Inc., San Diego, CA, USA). Reads for each sample were aligned to the reference genome for *Homo sapiens* (NCBI GRCh38 with decoys) with the Issac Aligner. Mutect2 [27] and Strelka2 [28] were used to call single-nucleotide variants (SNVs) and indels comparing the dermis and dura mater-derived cell lines to each other. Only consensus calls with a PASS value made by both callers were used. The variant allele fraction (VAF) distribution for each cell line was determined through the VCF files generated by Mutect2. The frequency of each of the 6 pyrimidine SNVs detected in each sample was determine through SigProfilerMatrixGenerator [29]. De novo extracted signatures were determined, reconstructed, and decomposed using known COSMIC single base substitution (SBS) signatures [30, 31]. De novo signatures were matched to a combination of COSMIC signatures and the number of mutations in each signature were determine for each sample.

### Annexin and Propidium Iodide Staining

From the same subject, paired dermis and dura mater-derived cell lines were thawed and 100,000 cells were set aside for annexin and propidium iodide (PI) staining using the Annexin V-FITC Apoptosis Staining/Detection Kit (Abcam, #ab14085) according to the manufacturer’s instructions. Briefly, cells were resuspended in 1X Binding Buffer and stained with 5 ul of Annexin V-FITC and 5 μl of PI (Texas Red) for five minuets at room temperature. Apoptosis and PI detection were determined utilizing an Attune NxT Flow Cytometer (Thermo Fisher Scientific).

### Statistical Analysis

Statistical analysis, t-test, linear regression, and odds ratios with a Fisher’s exact test were performed on GraphPad Prism 8. Immunofluorescence quantification was performed on ImageJ2. Mean and standard error were reported. P-values less than or equal to 0.05 were considered significantly different.

## Results

### The Freeze-Thaw Process Decreases Cell Outgrowth from Postmortem Dura Mater

To investigate the potential of postmortem dura mater as a viable source of cultured fibroblasts, we performed a systematic comparison of the ability to culture cells from two different sources, dermis and dura mater. Dermal fibroblasts were obtained from skin biopsies during life and dura mater was either processed shortly after autopsy (fresh) or from banked frozen dura mater (frozen).

For this comparison, we processed a total of 77 dermal biopsies for fibroblast cell banking [20]. These samples were obtained from subjects during life with a mean age of 53.6 ± 14.2 years and with more female subjects than male subjects, 62% and 38% respectively (Table 1). Most subjects were diagnosed with neurogenerative disease, either clinically or through neuropathological assessment (Supplemental Table S1). All processed dermal cell lines produced cell outgrowth, and cell lines were successfully expanded and banked in liquid nitrogen (Table 2). Thus, the generation of cell lines from dermal biopsies performed in living subjects was robust and consistent.

To compare the success rate of cell outgrowth from dura mater, we utilized the same isolation protocol as for dermal cell banking. We processed a total of 43 fresh dural cases, from subjects with a mean age of 74 ± 9.3 years, mean postmortem interval (PMI) of 11.1 ± 7.6 hours, and with a slightly greater number of male subjects than female subjects (53% and 47% respectively, Table 1). Most subjects were diagnosed with neurogenerative disease through neuropathological assessment (Supplemental Tables S1 and S2). Forty cases had cell outgrowth that led to successful banking (93%, Table 2). Furthermore, two dural cases that initially had no cell outgrowth were recovered by reprocessing tissue that was cryoprotected with DMSO and stored in liquid nitrogen post-autopsy. Three fresh dural cases displayed no outgrowth, despite extensive time in culture (~40 days). All dura mater-derived cell lines were negative for mycoplasma contamination (data not shown). In summary, cell outgrowth, generation, and banking from fresh dural tissue were comparable to dermal tissue.

In addition, we investigated the ability to culture cells from banked frozen dura mater. A total of 14 frozen cases were processed, with the mean age of subjects being 66 ± 8.5 years, mean PMI of 7.4 ± 2.2 hours, and male and female subjects were equally represented (Table 1). Only two frozen dural cases had cell growth that was sufficient for successful banking (14%, Table 2). Three additional dura mater samples had limited cell outgrowth, slow proliferation, and poor cell morphology and therefore could not be successfully expanded for banking. In an attempt to increase cell recovery from frozen dura mater, we utilized previously published alternative culture medias [18, 19] for seven frozen dura mater samples. Despite up to 40 to 70 days in culture, cell outgrowth using alternative media compositions and combinations did not consistently improve cell outgrowth. Additionally, we utilized paired dermis and dura mater-derived cells from the same subject for further interrogation. Notably, these cells were, by definition, isolated at two different points in time. The dermal biopsy occurred during life when the subject was at age 48 and the dura mater was frozen at the time of autopsy at age 51. These paired lines revealed an increase in apoptosis and cell death after thawing in the dura mater-derived cell line compared to the dermis-derived cell line (Supplementary Fig. 1). Overall, the fresh dural tissue showed significantly better cell outgrowth compared to frozen dural tissue (with an odds ratio of 80, 95% CI [10.71-407.9], *p* < 0.0001****, Fisher’s exact test).

### Dura Mater-Derived Cell Lines Exhibit Slower Outgrowth and Proliferation, as well as Abnormal Morphology

**Fig. 1:**
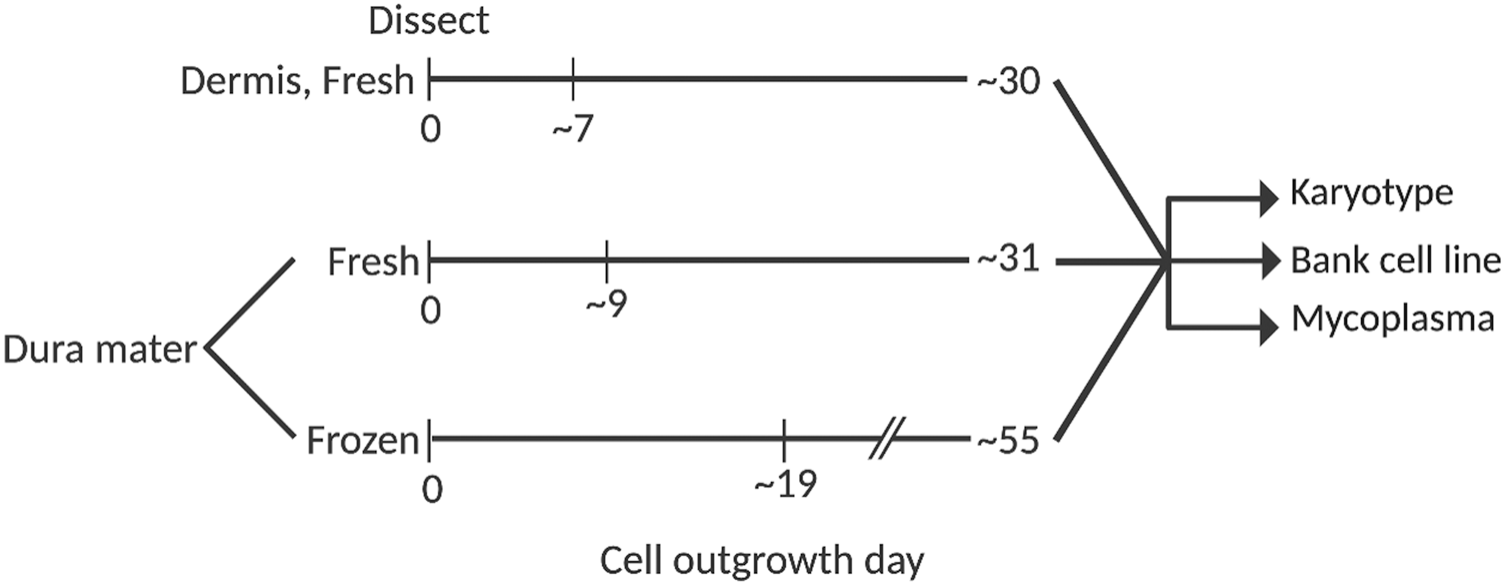
Timeline in days for the isolation, expansion, and banking of dermal cells from fresh dermis and dural cells from fresh and frozen dura mater. Cell outgrowth, proliferation, and banking from fresh and frozen dural tissue were delayed compared to dermal tissue. Successfully banked cell lines were karyotyped and tested for mycoplasma

As dermis and dura mater-derived cell lines were cultured and banked, differences in cell growth, morphology, and proliferation rates became apparent. From dermal tissue, cell outgrowth was typically observed by day seven post-dissection and an adequate number of cells were expanded for cryopreservation after thirty days. In contrast, cell outgrowth from fresh dura mater was observed approximately nine days post-dissection and cells were cryopreserved after thirty-one days. Finally, cells derived from frozen dural tissue required 19 days for outgrowth and 55 days to be expanded for cryopreservation (Fig. 1). Thus, initial cell outgrowth and banking from dural tissue took longer compared to dermal tissue.

**Fig. 2:**
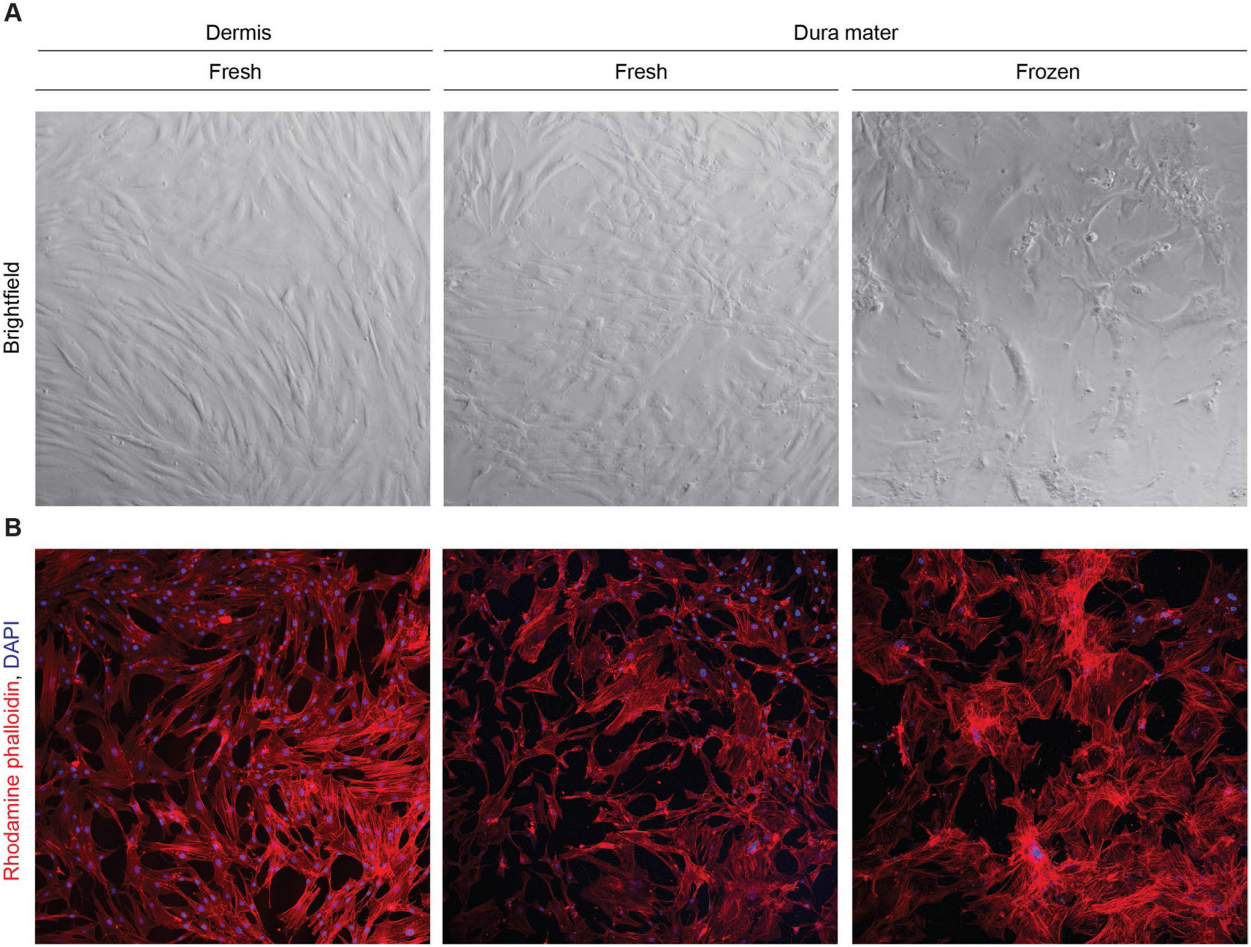
Dural cells displayed reduced cell outgrowth with larger cell body morphology than cultured dermal cells. **A** Dermal cells exhibited classical fibroblast morphology. In contrast, dural cells were enlarged, variable, and had disorganized alignment. Representative brightfield images are shown for each cell type and condition with a 5x objective **B** Cells were stained with rhodamine phalloidin (red) and DAPI (blue), reflecting differences in actin filament arrangement between dermal and dural cells. Representative immunofluorescent images are shown for each cell type and condition with a 10x objective, performed in triplicates

Differences in cell morphology between the dermis and dura mater-derived cell lines were also noted, where paired dermis and frozen dura mater-derived cells from the same subject were utilized. Brightfield images demonstrated that dermis-derived cells exhibited classical fibroblast morphology, with long, spiny cell bodies that grew in tightly packed formation. Dural cells derived from fresh dura mater appeared to have an intermediate phenotype, with some spindle-shaped cell bodies that were densely packed and others with a more cobblestone shape and disorganized alignment. In contrast, dural cells derived from frozen dura mater displayed abnormal morphological features, such as enlarged cell bodies, decreased uniformity, and decreased cell density (Fig. 2A). Rhodamine phalloidin staining for actin filaments further illustrated the decreased organization of dura mater-derived cell lines (Fig. 2B). Taken together, these observations show differences in cell morphology and arrangement between dermis and dura mater-derived cells, suggesting potential abnormalities in the dura mater-derived cell lines.

**Fig. 3:**
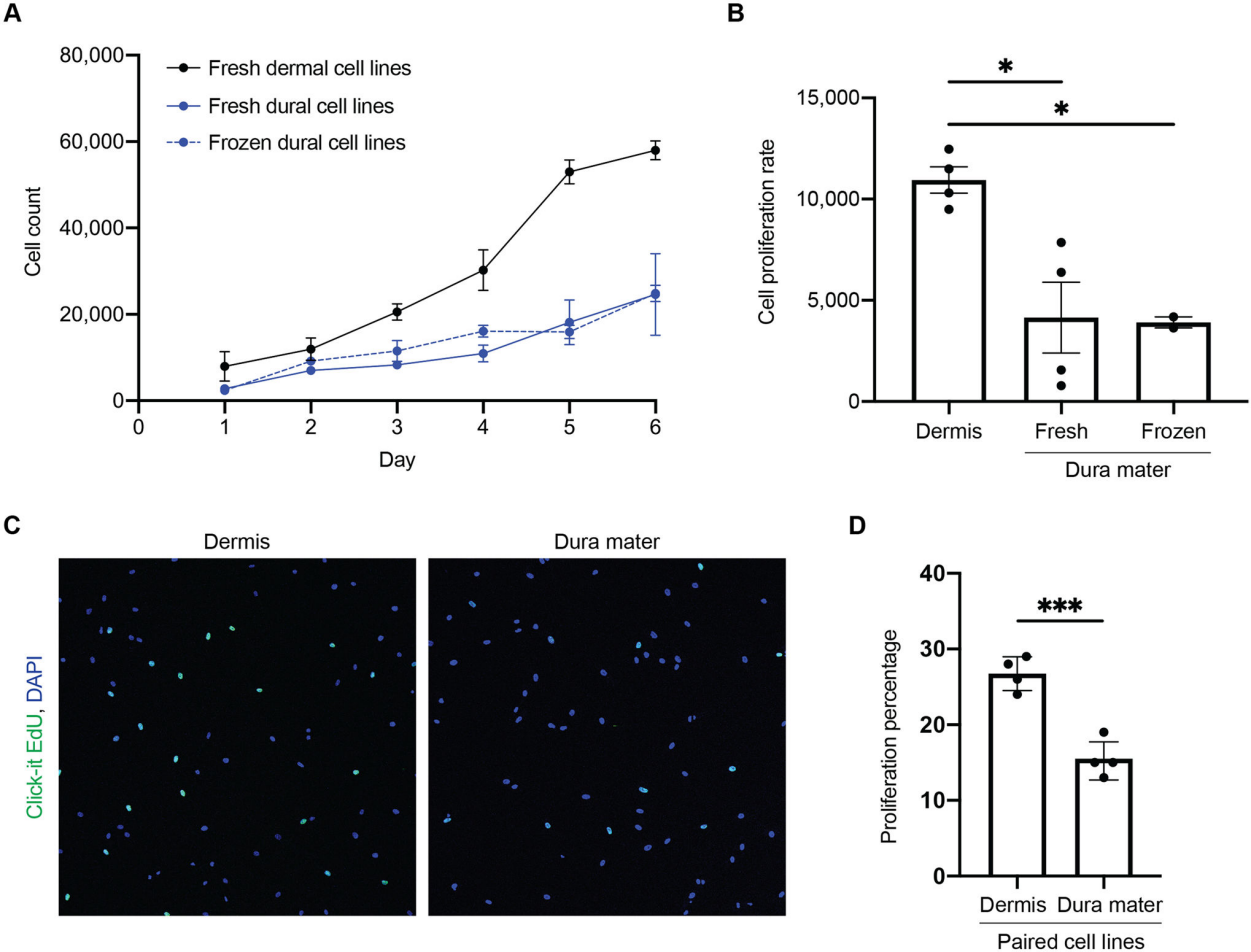
Differences in cell proliferation were observed between cells derived from dermis and dura mater. **A** The total number of cells were counted for a span of six days for four dermal cell lines (black), four fresh dural cell lines (solid blue), and two frozen dural cell lines (dotted blue), performed in triplicates. **B** Cell proliferation rates for dermal and dural cell lines are shown. There was a significant decrease in cell proliferation in both frozen and fresh dural cell lines compared to the dermal cell lines (One-way ANOVA, * *p* < 0.05). **C** Proliferation assay with Click-it EdU (green) and DAPI (blue) for paired dermal (left) and dural (right) cell lines from the same subject, performed in quadruplicates. **D** Percentage of EdU positive cells of the total DAPI positive cells. There was a significant decrease in cell proliferation in the dural cell line compared to the paired dermal cell line (unpaired t-test, *** *p* < 0.0005)

Next, we documented proliferation rates of these cell lines. Compared to dura mater-derived cell lines, dermis-derived cell lines resulted in more total cells counted over the span of six days (Fig. 3A). Proliferation rate of dura mater-derived cell lines was significantly decreased compared to dermis-derived cell lines (Fig. 3B). Overall, dermis-derived cell lines were observed to proliferate about three times faster than dura mater-derived cell lines. Furthermore, the incorporation of EdU in the paired dermis and dura mater-derived cell lines from the same subject revealed a significant decrease in DNA synthesis in the dura mater-derived cell line compared to dermis-derived cell line (Fig. 3C, D).

### Dura Mater-Derived Cell Lines Exhibit Chromosomal Abnormalities

As an important quality control in cell banking, we performed chromosomal karyotyping to assess gross genetic changes or anomalies in the derived cell lines. From the thirty-six dermis-derived cell lines that underwent karyotype analysis, thirty-one exhibited a normal karyotype (86%) and five cell lines exhibited an abnormal karyotype (14%). In contrast, from the twenty-nine fresh dura mater-derived cell lines that underwent karyotyping, twelve cell lines exhibited a normal karyotype (41%), thirteen cell lines exhibited an abnormal karyotype (45%), and four cell lines exhibited an unstable karyotype (14%). Of note, the two cell lines derived from frozen dura mater that were successfully banked both exhibited normal karyotypes (Table 2). Overall, dura mater-derived cell lines revealed significantly more chromosomal instability than dermis-derived cell lines.

We wondered whether sex of the donor affected karyotype. Interestingly, from the 13 dura mater-derived cell lines that exhibited abnormal karyotypes, 12 cell lines were derived from male subjects (92%). Furthermore, the majority of male-derived cell lines with chromosomal abnormalities exhibited the clonal loss of chromosome Y (LOY). There were eight cell lines that exhibited only LOY, three cell lines that exhibited LOY with additional autosomal abnormalities, and one cell line that exhibited trisomy in chromosome 7 (Table 3). In summary, there were more chromosomal abnormalities in male-derived dural cell lines compared to female-derived dural cell lines (with an odds ratio of 16.8, 95% CI [1.615-201.2], *p* = 0.0112*, Fisher’s exact test).

### Dura Mater-Derived Cell Lines Fail to Express Key Fibroblast Markers

**Fig. 4:**
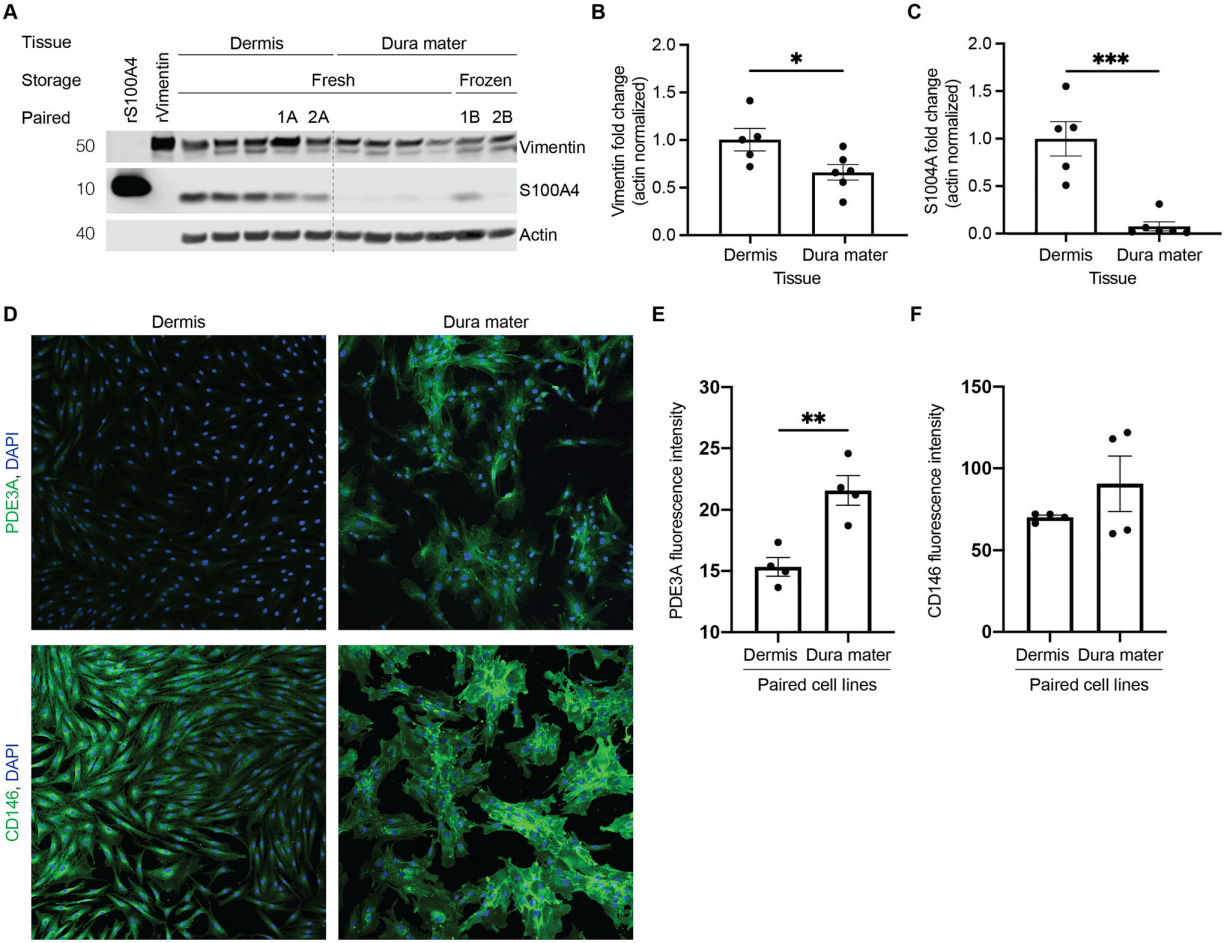
Protein expression differences in fibroblast markers were observed between dermal and dural cell lines. **A** Cell lysates from five dermal and six dural cell lines were immunoblotted against Vimentin and S100A4. Tissue indicates from which source the cells were derived from, storage indicates whether tissue underwent frozen or fresh storage conditions before processing, and paired indicates cell lines derived from the same subject (2 cases, lanes 1A/1B and 2A/2B). rVimentin and rS100A4 were recombinant proteins that were used as positive controls for their respective antibodies. **B** There was a significant decrease in vimentin protein levels in dural cell lines relative to the dermal cell lines (normalized to actin, unpaired t-test, * *p* < 0.05). **C** There was a significant decrease in S100A4 protein levels in dural cell lines relative to the dermal cell lines (normalized to actin, unpaired t-test, *** *p*< 0.0005). **D** Immunofluorescence for PDE3A (green, top panel), CD146 (green, bottom panel), and DAPI (blue) for paired dermal (left) and dural (right) cell lines from the same subject, performed in quadruplicates. **E** Quantification of mean PDE3A fluorescence intensity with a significant increase in the dural cell line compared to the paired dermal cell line (unpaired t-test, ** *p* < 0.005). **F** Quantification of mean CD146 fluorescence intensity in the paired dermal and dural cell lines

Given the striking differences in morphology, proliferation rate, and karyotype between dermal and dural cells, we next questioned the cellular identity of dura mater-derived cell lines (Supplementary Table S3). We assessed cell identity by immunostaining against two classical markers of fibroblast identity, the intermediate filament protein vimentin and the fibroblast-specific protein 1 (FSP1 or S100A4), a member of the S100 calcium binding family [32] (Fig. 4A). While dura mater-derived cells expressed vimentin, albeit at lower levels, they lacked expression of S100A4 (Fig. 4A-C). In this analysis, we were able to compare protein expression in two pairs of dermis and dura mater-derived cells from the same subjects (Fig. 4A, lanes 1A/1B and 2A/2B). Notably, in these paired cell lines, levels of the S100A4 fibroblast marker appeared decreased in the cells derived from postmortem dura mater compared to cells derived from dermis. Together, these results raised the question of whether dura mater-derived cells were of a fibroblast lineage.

### Dermis and Dura Mater-Derived Cells from the Same Subject Exhibit Highly Divergent Gene Expression Profiles Suggesting a Mural Origin for Dura Mater-Derived Cells

**Fig. 5:**
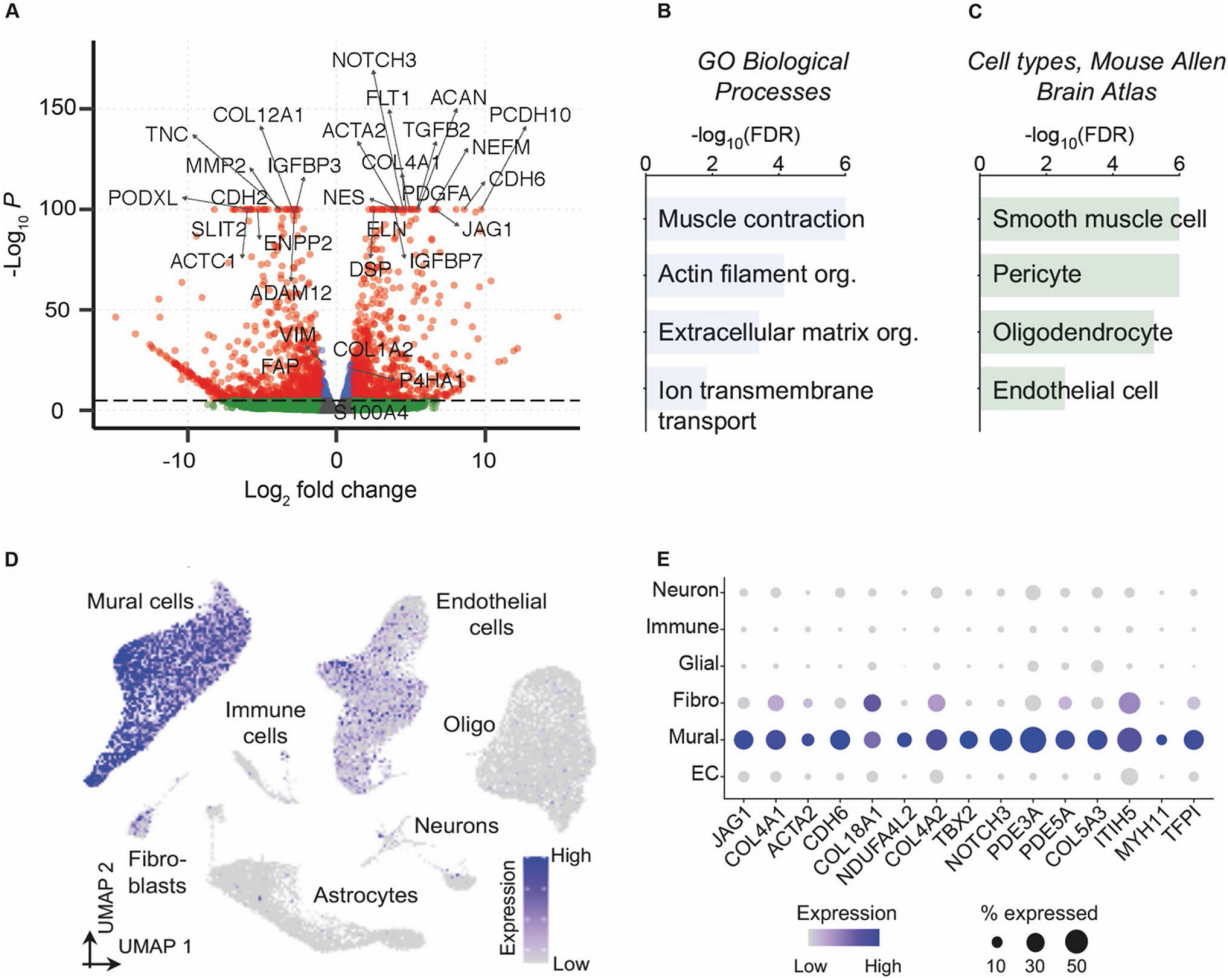
Cells cultured from human postmortem dura mater adopt mural cell gene expression patterns. **A** Volcano plot of differentially expressed genes. Red dots denote differentially expressed genes, blue dots denote genes with expression differences of −Log_10_*P* between −1 and 1, and the green dots denote non-significantly expressed genes. **B** GO Biological Processes significantly enriched amongst genes upregulated in dural cells. **C** Dural cells enriched cell types from the mouse Allen Brain Atlas. Top enriched pathways and cell types with a false discovery rate (FDR) <0.05 are shown (Benjamini-Hochberg correction). **D** UMAP feature plot mapping the combined expression of the top 50 genes upregulated in dural cells compared to vascular-enriched nuclei from human postmortem brains. **E** Example of 15 genes from (D), demonstrating human brain mural-specific expression of genes upregulated in dural cells

To better understand how transcriptional profiles compared between cells cultured from dermal and dural tissues and to gain insight into the cell lineage of dura mater-derived cells, we performed RNA-sequencing. For this analysis, we utilized the paired dermis and dura mater-derived cells from the same subject. RNA-sequencing analysis revealed approximately 3000 genes that were significantly differentially expressed between the dermis and dura mater-derived cell lines. Genes upregulated in dura mater-derived cells included *ACTA2* (Smooth muscle actin), *NOTCH3* (Notch 3), and *COL4A1* (Component of type IV collagen) and significantly downregulated genes included *COL15A1* (Collagen type XV alpha 1 chain) and *FBLN1* (Fibulin 1) (Fig. 5A, Additional File 1). These genes implicate differences in extracellular matrix, structure, filaments, and signaling. Given the number of differentially expressed genes, we further investigated molecular pathways that differed between the dermis and dura mater-derived cell lines. Enriched Gene Ontology (GO) Biological Processes included categories such as “muscle contraction”, “actin filament organization”, “extracellular matrix organization”, and “ion transmembrane transport” in dura mater-derived cells (Fig. 5B).

Even knowing that developing dura mater contains several fibroblast types that exhibit distinctive transcriptional profiles [33], the RNA-sequencing results fueled concern that our dura mater-derived cells may not be of purely fibroblast identity. In fact, the perivascular niche of dura mater consists of several different cell types, including smooth muscle, pericytes, oligodendrocytes, fibroblasts, and endothelial cells [34]. Therefore, we compared our RNA sequencing data to transcriptional profiles from vascular enriched nuclei extracted from human postmortem brain tissue [25]. When mapped to the mouse single cell Allen Brain Atlas [35], this comparison revealed that dura mater-derived cells were enriched in mural smooth muscle cells and pericytes—and to a lesser extent, oligodendrocytes and endothelial cells (Fig. 5C). Furthermore, Uniform Manifold Approximation and Projection (UMAP) mapping of the top 50 upregulated genes in dura mater-derived cells were highly mural cell-specific when compared to vascular-enriched nuclei extracted directly from human postmortem brains without culturing (Fig. 5D) [25]. This shift in dura mater-derived cells towards a mural cell lineage is exemplified by their specific expression of smooth muscle cell and pericyte marker genes such as *JAG1* (Jagged-1) and *ACTA2*, (Smooth muscle actin) and *PDE3A* (Phosphodiesterase 3A) and *TFPI* (Tissue factor pathway inhibitor) (Fig. 5E). Furthermore, the PDE3A pericyte marker [25] immunofluorescence expression was significantly increased in the dura mater-derived cell line compared to the paired dermis-derived cell line (Fig. 4D, E). CD146 smooth muscle cell marker [25] immunofluorescence expression had an increasing trend in the dura mater-derived cell line compared to the paired dermis-derived cell line (Fig. 4D, F). Together, these results suggest that the dura mater-derived cells do not correspond to fibroblasts or any single cell type, but rather adopt a broad mural cell identity.

### Dermis and Dura Mater-Derived Cells from the Same Subject Reveal Different Mutation Signatures

**Fig. 6:**
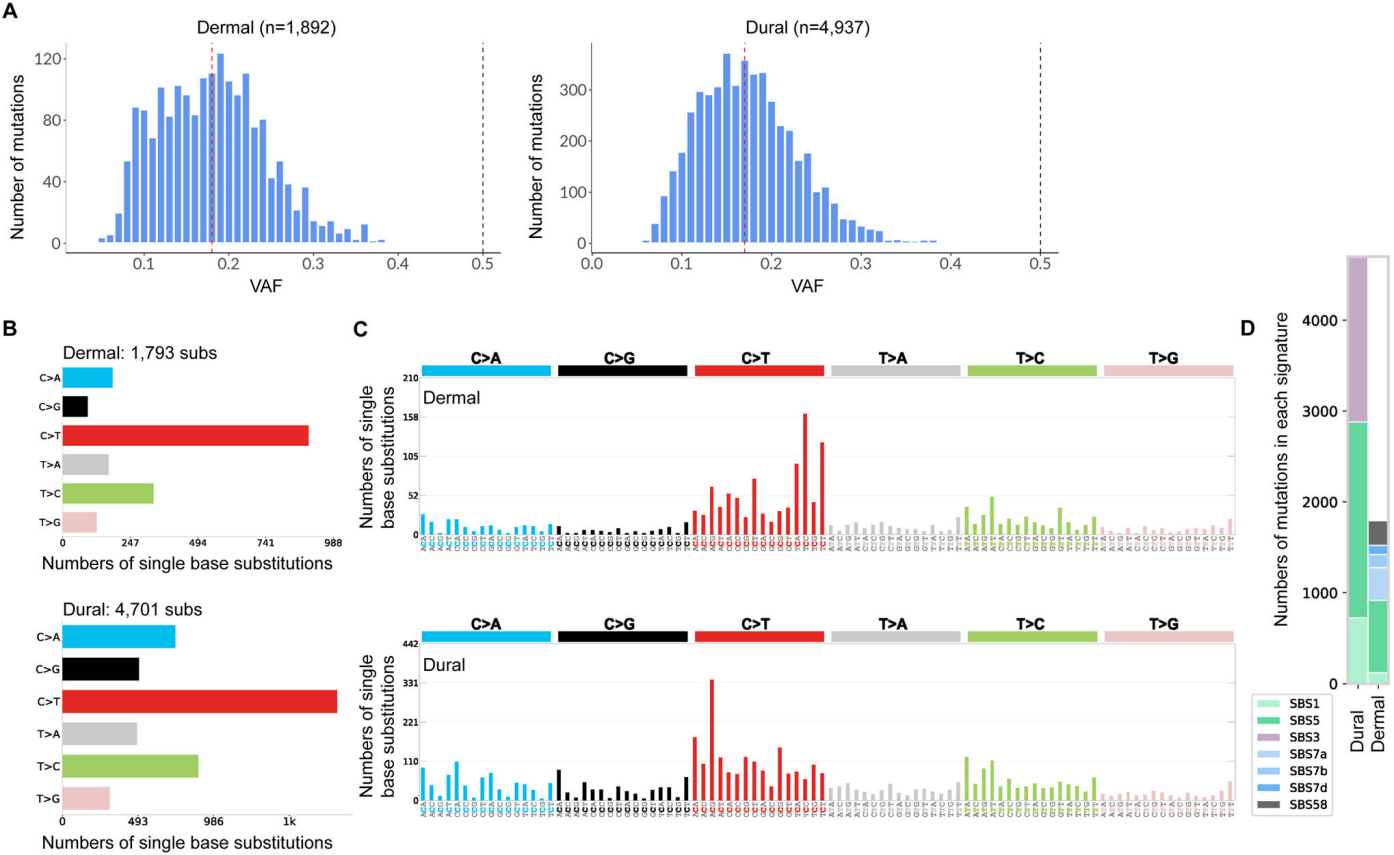
Cells cultured from human postmortem dura mater exhibit enhanced numbers and a differential pattern of somatic mutations compared to dermal cells. **A** VAF distribution of discovered mutations from dermal and dural cell lines from the same subject. The red dashed line represents the median VAF and the black dashed line represents 50% VAF. **B** The frequency of each of the 6 pyrimidine SNVs and their complements called in each cell line. **C** The frequency of the pyrimidine SNVs that are separated into 96 possible combinations (4 starting nucleotides × 6 pyrimidine variants × 4 ending nucleotides). COSMIC SBS signatures are identified using these 96 different contexts. **D** COSMIC SBS signatures that are decomposed using contexts in (C). Bar graph shows how many mutation calls in each cell line that contribute to each of the decomposed COSMIC signatures

To better understand how postmortem cell culturing affects accumulation of somatic mutations, the same pair of dermis and dura mater-derived cell lines underwent WGS. The median VAF for the dermis-derived cell line was 0.18 and for the dura mater-derived cell line, 0.17 (Fig. 6A), suggesting that both cell lines were multi-clonal in nature. In contrast, one expects the majority of mutations in clonal lines to be one of two haplotypes, resulting with a VAF around 50%. When whole genome sequences from the two lines were compared to each other, dura mater-derived cells exhibited substantially more variant calls than dermal-derived cells (4937 versus 1892 respectively).

The number of single base substitutions were evaluated for each of the six pyrimidine subtypes (C > A, C > G, C > T, T > A, T > C, and T > G) and their complements. While C to T base substitution was the most abundant in both cell lines, fractions of the other base substitutions were higher in the dura mater-derived cell line than in the dermis-derived cell line, suggesting differences in mutational processes between those two cell types (Fig. 6B-C). Next, mutational signatures of the cell lines were evaluated based on somatic mutations found in cancer genomes [30, 36]. The de novo mutation signatures found in each cell line were reconstructed and matched to a unique combination of known COSMIC signatures (Fig. 6D). SBS1 and SBS5 COSMIC signatures were observed in both cell lines, with lesser SBS1 mutations in the dermis-derived cell line. SBS1 is known to be related to deamination of 5-methylcytosine to thymine which generates G:T mismatches in double stranded DNA [30]. Mutations that contribute to SBS5 are the most abundant in both cell lines, but its etiology is unknown. Both SBS1 and SBS5 signatures are known to be clock-like in that the number of mutations in cells correlates with the age of the individual [30]. Unique contributing mutation signatures for the dermis-derived cell lines were like those found in cancers of the skin from sun exposure (SBS7a, SBS7b, and SBS7d), which can be expected from the dermis tissue that was collected. In contrast, the sun exposure mutation signatures were not found in the dura mater-derived cell line. Instead, the dura mater-derived cell line had a unique mutation signature associated with failure to repair homologous recombination-based DNA damage (SBS3). Finally, a subset of mutations was found in the dermal-derived cell line reflecting a potential sequencing artefact (SBS58) (Fig. 6D). Overall, these analyses revealed divergence of mutations processed in the two cell types used to derive the lines.

## Discussion

Fibroblast-like cells isolated from dura mater have been generated and reprogrammed into iPSCs and differentiated into neurons [17-19]. Postmortem dura mater-derived cells are advantageous when no other cell source is available. With the hope of advancing dura mater-derived cells as a potential cell source for precision medicine applications, we undertook a systematic comparison between cells isolated from dermal biopsies during life versus postmortem dura mater, either immediately after autopsy or after a period of being frozen. Earlier studies have characterized dura mater cells either collected perioperatively [18] or from postmortem tissue [17, 19, 37] In the latter cases, the dura mater cells were compared to cells derived from postmortem scalp dermis [17, 19] In contrast, this study compared postmortem dural cells to dermal fibroblasts collected during life. A comparison of the study materials, conditions used, results reported here, and prior dura mater cell studies can be found in ‘Additional file 2’. Our results revealed substantive differences between dermis and dura mater-derived cell lines in cell outgrowth, proliferation, morphology, karyotype, protein expression, transcriptional profiles, and genome sequencing.

Mapping the transcriptomes of our dura mater-derived cells to those of nuclei profiled directly from human postmortem brains [25] confirmed that dura mater-derived cells are transcriptionally distinct from dermis-derived fibroblasts. In fact, the dura mater-derived cells do not correspond to only one cell type in vitro, but rather consist of a mixed population or possibly cells in an intermediate, transitioning cell state. Interestingly, the RNA-sequencing analysis identified a clear down-regulation of fibroblast genes and a strong upregulation of genes corresponding to mural cells, an umbrella term for vascular smooth muscle cells and pericytes [38]. Our comparison thus suggests an unexpected lineage shift of dura mater-derived cells towards a mural cell lineage.

The contrasts between our results and those of other groups may be attributed to technical differences. For example, Sproul et al. reported a higher success rate of fibroblast outgrowth from dura mater, which was processed by freezing in a liquid nitrogen vapor sandwich, with the tissue first chilled between aluminum plates and then placed in liquid nitrogen vapor for five minutes [19]. In comparison, we froze dura mater using a dry ice sandwich method, which is significantly warmer than liquid nitrogen vapor [39]. With these freezing conditions and the lack of a cryoprotectant reagent to prevent ice damage, the frozen dura mater and dural cells may have been damaged, resulting in the unsuccessful establishment of working cell lines or altered morphology. Therefore, careful storage precautions should be taken into consideration while freezing dura mater without a cryoprotectant reagent.

Cell outgrowth issues were ameliorated in dura mater that was processed fresh, without being frozen. Despite this, cell lines from fresh dura mater still contained a higher percentage of chromosomal abnormalities when compared to dermal fibroblasts. This observation could stem from several possibilities, including postmortem tissue source, age of subject, PMI, or more advanced stage of neurodegenerative disease. A significant proportion of the chromosomal abnormalities in dura mater-derived cell lines were from male cases that exhibited LOY. Interestingly, it has been previously reported that LOY in blood cells was associated in aging men with diseases, such as cancer and Alzheimer’s disease (AD) [40]. This may suggest that postmortem dura mater-derived cell lines from subjects with neurodegenerative diseases have chromosomal instability in vitro.

Sproul et al. also previously reported LOY in a dura mater-derived iPSC line which they attributed to in vitro culturing because the Y chromosome was present in the original frozen dura mater [19]. This was determined through *AMG* (Amelogenin) gene amplification as the amplicon will differ in size depending on its chromosome location [19]. Sex chromosome abnormalities have also been described in iPSCs derived from cells isolated from leptomeninges [41]. Our findings are consistent with these earlier studies. Interestingly, dura mater obtained intraoperatively resulted in an iPSC line with a normal male karyotype [18]. This suggests that karyotype and other genetic abnormalities may originate from the postmortem period. In fact, it has been previously reported that human stem cells with chromosomal abnormalities exhibit a cellular growth advantage in vitro, which may track with the high rate of karyotype abnormality in the dura mater-derived cell lines of our study. The mechanisms of chromosomal abnormalities remain unclear [42].

While karyotype abnormalities appear to be a significant concern for dura mater-derived cells, dural cell lines with a normal karyotype nonetheless did not exhibit a clear fibroblast identity based on protein markers and RNA-sequencing profile. Expression differences in cell lines derived from dermis and dura mater may be due to the different tissue types and anatomical locations, as it has been reported for fibroblasts isolated from different body regions such as the arm or thigh [43, 44]. Others have reported a lack or decrease of S100 family protein expression in dura mater-derived cells [15, 19, 37], similar to what we have shown here. However, since our RNA-sequencing analysis suggests that the dura mater-derived cells are shifting towards a more mural cell lineage rather than a fibroblast one, it is more likely that these differences in expression and morphology are due to the composition and source of the tissue biopsy itself. Immunofluorescence of pericyte and smooth muscle cell markers in the dura mater-derived cell line further corroborates differences in cell type identity. Assuming that the dura mater-derived cells contain smooth muscle cells and pericytes, we asked if these can be reprogrammed into iPCSs. A review of the literature reveals that pericytes can be reprogrammed into iPSCs [45]. However, in Karow et al., pericytes were derived perioperatively rather than postmortem sources and thus do not risk the high rate of karyotype abnormality.

The WGS results revealed additional differences between dermal and postmortem dural cells: (1) the number of somatic small variants are higher in dura mater-derived cells, (2) some mutational signatures in the dermis and dura mater-derived cells are different and (3) the dura mater-derived cells contain mutations reflecting defective DNA damage repair. The number of variants in both cell lines are consistent with that previously noted by Abyzov et al., who estimated approximately one thousand mosaic SNVs can be found in a single fibroblast cell from children and then subsequently seen in the derived iPSCs [46]. Larger mutation counts in dura mater-derived cells, even if only considering mutations related to age associated signatures SBS1 and SBS5, may reflect overall higher mutability of these cells in vivo. Alternatively, the hypoxia that necessarily precedes the recovery of postmortem dural cells predisposed to acquisition of more mutations in vitro. This alternative is consistent with both in the high degree of karyotypic abnormalities (Table 3) and the DNA damage mutation signature (Fig. 6D) of these cells.

If postmortem dura mater-derived cells have elevated rates of gene mutations and variants, they may not accurately or reproducibly reflect normal biological or pathobiological processes. Ultimately, they may be deemed inappropriate for disease modeling. Our findings suggest that the use of dura mater-derived cells for direct differentiation or reprogramming should proceed with caution.

## Conclusions

We aimed to assess the possibility of utilizing postmortem dura mater, from either frozen or fresh storage conditions, as a source of in vitro disease cell models. Contrary to our expectations, this study revealed multiple phenotypic, karyotypic, transcriptional, and genomic differences between dermis and dura mater-derived cells, even when those cells originated from the same subject. Furthermore, the dura mater-derived cells were of mixed mural rather than a purely fibroblast origin. Thus, dura mater-derived cells may not be appropriate for modeling of normal or abnormal biological processes without careful consideration of karyotype, gene sequencing, and cell type identification.

## Supplementary Material

suppl. file 1

suppl. file 3

suppl. file 2

## Figures and Tables

**Table 1:** Demographics of dermal and dural cases

	Dermis	Dura mater	
Mean	Fresh	Fresh	Frozen
Number of cases	77	43	14
Age (years)	53.6 ± 14.2	74 ± 9.3	66 ± 8.5
PMI (hours)	–	11.1 ± 7.6	7.4 ± 2.2
Sex (M/F)	29/48	23/20	7/7

**Table 2:** Successful cell outgrowth and karyotype (KT) analysis of dermal and dural cases

	Dermis	Dura mater	
	Fresh	Fresh	Frozen
Outgrowth	77/77 (100%)	40/43 (93%)	2/14 (14%)
Normal KT	31/36 (86%)	12/29 (41%)	2/2 (100%)
Abnormal KT	5/36 (14%)	13/29 (45%)	0/2 (0%)
Unstable KT	0/36 (0%)	4/29 (14%)	0/2 (0%)

**Table 3:** Chromosomal abnormalities of dural cell lines from fresh dura mater

	Male (n = 12)	Female (n = 1)
Trisomy	1	1
LOY	8	–
LOY with autosomal abnormalities	3	–

*LOY* Loss of chromosome Y

## Data Availability

Cell lines are available upon reasonable request through the Memory and Aging Center at the University of California, San Francisco. A detailed dura mater-derived cell culture protocol can be found at protocols.io: dx.doi.org/10.17504/protocols.io.8m2hu8e. RNA-sequencing data from this study has been deposited to Gene Expression Omnibus (GSE178933).

## Abbreviations

AD: alzheimer’s disease
ADNC: alzheimer’s disease neuropathologic change
AGD: argyrophilic grain disease
ALS: amyotrophic lateral sclerosis
BSA: bovine serum albumin
CTE: chronic traumatic encephalopathy
DAPI: 4′,6-diamidino-2-phenylindole
DMEM: dulbecco’s modified eagle medium
DMSO: dimethyl sulfoxide
DPBS: dulbecco’s phosphate buffered saline
EDU: 5-ethynyl-2′-deoxyuridine
FBS: fetal bovine serum
FDR: false discovery rate
FTLD: frontotemporal lobar degeneration
GO: gene ontology
iNeurons: induced neurons
iPSCs: induced pluripotent stem cells
KT: karyotype
LBD: lewy body dementia
LOY: loss of chromosome Y
NEAA: non-essential amino acid
PI: propidium iodide
PMI: postmortem interval
rS100A4: recombinant S100A4
rVimentin: recombinant vimentin
S100A4/FSP1: fibroblast-specific protein 1
SBS: single base substitution
SNV: single-nucleotide variants
TDP-43: TAR DNA-binding protein 43
TRITC: tetramethyl-rhodamine
UMAP: uniform manifold approximation and projection
VAF: variant allele fraction
WGS: whole genome sequencing
