# Supplementary material for: Postmortem Human Dura Mater Cells Exhibit Phenotypic, Transcriptomic and Genetic Abnormalities that Impact their Use for Disease Modeling
Source: Stem Cell Rev Rep. Author manuscript; Available in PMC 2022 Dec 1. (PMC9622518; doi:10.1007/s12015-022-10416-x)
Supplement: suppl. file 1 [file NIHMS1832445-supplement-suppl__file_1.docx]

**Additional files**

1. File name: Additional file 1
   1. File format: xlsx
   2. Title of data: Dural versus dermal gene logFC
   3. Description of data: Genes log fold change between dural versus dermal cells
2. File name: Additional file 2
   1. File format: xlsx
   2. Title of data: Comparison table of dura mater papers
   3. Description of data: Comparison table of selected papers that derived cells from dura mater

**Supplemental Tables**

**Table S1** Diagnosis of dermal and dural cases

|  | **Dermis** | **Dura mater** | |
| --- | --- | --- | --- |
|  | **Fresh (n = 77)** | **Fresh (n = 43)** | **Frozen (n = 14)** |
| Neuropathological diagnoses of disease | 8 (10%) | 38 (88%) | 14 (100%) |
| Clinical diagnoses of disease | 51 (66%) | - | - |
| Clinically normal | 14 (18%) | 5 (12%) | - |
| Biospecimen only | 4 (5%) | - | - |

**Table S2** Neuropathological diagnosis of all dural cases

| **Neuropathological diagnosis** | **No. of cases** |
| --- | --- |
| AD | 13 |
| ADNC | 6 |
| AGD | 1 |
| ALS-TDP-43 | 1 |
| CTE | 1 |
| Chronic Wernicke-Korsakoff Encephalopathy | 1 |
| FTLD-TAU (PSP, CBD, Pick's) | 19 |
| FTLD-TDP-43 (Type A, B, Unclassifiable) | 6 |
| LBD | 3 |
| Motor and sensory neuropathy | 1 |
| Clinically normal | 5 |

AD = Alzheimer's disease, ADNC = Alzheimer's disease neuropathologic change, AGD = Argyrophilic grain disease, ALS = Amyotrophic lateral sclerosis, CTE = Chronic traumatic encephalopathy, TDP-43 = TAR DNA-binding protein 43, FTLD = Frontotemporal lobar degeneration, LBD = Lewy body dementia

**Table S3** Selected dermal and dural cell lines for western blot analysis

| **Lane** | **Derived tissue** | **Tissue arrival** | **Age at biopsy** | **Age at death** | **PMI** | **Sex** |
| --- | --- | --- | --- | --- | --- | --- |
| 1 | Dermis | Fresh | 72 | - | - | M |
| 2 | Dermis | Fresh | 52 | - | - | F |
| 3 | Dermis | Fresh | 67 | - | - | F |
| 4^1A^ | Dermis | Fresh | 48 | - | - | F |
| 5^2A^ | Dermis | Fresh | 65 | - | - | F |
| 6 | Dura mater | Fresh | - | 72 | 28.2 | M |
| 7 | Dura mater | Fresh | - | 69 | 11.6 | M |
| 8 | Dura mater | Fresh | - | 60 | 20.5 | F |
| 9 | Dura mater | Fresh | - | 82 | 9.6 | F |
| 10^1B^ | Dura mater | Frozen | - | 51 | 5.4 | F |
| 11^2B^ | Dura mater | Frozen | - | 66 | 7.7 | F |

Dermal cell line 1A and dural cell line 1B are from the same subject.

Dermal cell line 2A and dural cell line 2B are from the same subject.

**Supplemental Figure**

**
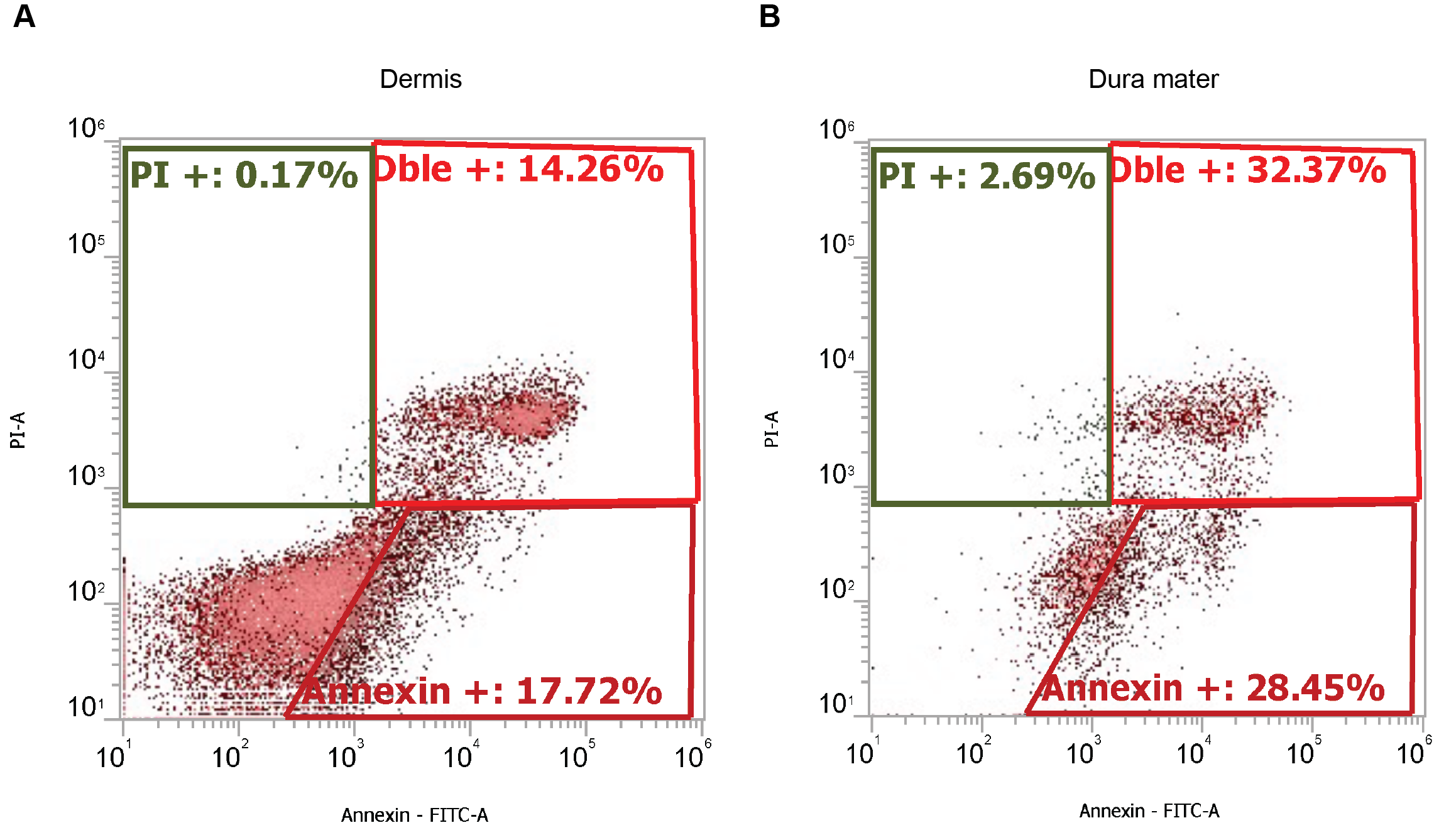
**

**Supplemental Figure 1.** Differences in **annexin and PI staining** were observed between paired dermal and dural cell lines from the same subject. **A Flow cytometry analysis of annexin (dark red) and PI (green) staining of the paired dermal cell lines after thawing. Dble indicates dual staining. B Flow cytometry analysis of annexin (dark red) and PI (green) staining of the paired dural cell lines after thawing. Dble indicates dual staining.**
